# 蛋氨酸代谢的预后模型构建及其在弥漫大B细胞淋巴瘤进展中的作用

**DOI:** 10.3760/cma.j.cn121090-20251219-00603

**Published:** 2026-05

**Authors:** 博雅 王, 梅凤 顾, 建勇 李, 紫娟 伍

**Affiliations:** 南京医科大学第一附属医院，江苏省人民医院血液科淋巴瘤中心，南京 210029 Lymphoma Center, Department of Hematology, the First Affiliated Hospital of Nanjing Medical University, Jiangsu Province Hospital, Nanjing 210029, China

**Keywords:** 淋巴瘤，大B细胞，弥漫性, 蛋氨酸代谢, 预后模型, 蛋氨酸限制, Lymphoma, large B-cell, diffuse, Methionine metabolism, Prognostic model, Methionine restriction

## Abstract

**目的:**

构建基于蛋氨酸代谢相关基因的预后风险模型，探究其在弥漫大B细胞淋巴瘤（DLBCL）中的预后评估价值以及蛋氨酸代谢异常在DLBCL疾病进展中的调控作用。

**方法:**

从GEO数据集（GSE10846）获取DLBCL患者预后信息及蛋氨酸代谢基因表达数据，采用Lasso回归分析和Cox回归分析筛选核心预后基因并构建风险模型，以GSE11318和GSE181063数据集验证模型可靠性。通过基因本体（GO）、京都基因与基因组百科全书（KEGG）通路分析和基因集富集分析（GSEA）解析模型潜在生物学功能。体外培养DLBCL细胞株U2932和SUDHL-4，设置正常培养组（对照组）、蛋氨酸剥夺组、蛋氨酸剥夺+蛋氨酸补充组（蛋氨酸补充组）、蛋氨酸剥夺+S-腺苷蛋氨酸（SAM）补充组（SAM补充组），采用液相色谱-串联质谱（LC-MS）检测细胞内蛋氨酸、SAM浓度，CCK-8实验和流式细胞术分别检测细胞增殖活性与凋亡水平。构建U2932细胞荷瘤NOD-SCID小鼠模型，分为常规饮食对照组（5只）、蛋氨酸限制性饮食组（5只），动态监测肿瘤生长情况。

**结果:**

从33个蛋氨酸代谢相关基因中筛选出8个核心基因构建预后风险模型，训练集Kaplan-Meier分析显示，高风险组患者总生存较低风险组差（*HR*＝2.50，95％ *CI*：1.84～3.39，*P*<0.001），验证集GSE11318（*HR*＝1.89，95％ *CI*：1.30～2.75，*P*<0.001）、GSE181063（*HR*＝1.39，95％ *CI*：1.20～1.62，*P*<0.001）验证了模型的稳定预后预测能力。体外实验显示，与对照组相比，蛋氨酸剥夺组细胞内蛋氨酸、SAM浓度降低（*P*值均<0.001），细胞增殖完全受抑且凋亡水平升高（*P*值均<0.001）；与蛋氨酸剥夺组相比，蛋氨酸补充组、SAM补充组细胞凋亡水平恢复（*P*值均<0.001）。动物实验显示，与对照相比，蛋氨酸限制性饮食组小鼠肿瘤体积［（438.3±179.1）mm^3^对（1 451.0±464.9）mm^3^，*P*＝0.002］、肿瘤重量［（0.3±0.1）g对（1.1±0.4）g，*P*＝0.004］均降低。

**结论:**

基于蛋氨酸代谢相关基因构建的预后风险模型具有DLBCL预后预测潜力，且蛋氨酸代谢异常可调控DLBCL细胞增殖与凋亡，为靶向蛋氨酸代谢的DLBCL干预研究提供了实验依据。

## Abstract (en)

**Objective:**

To construct a prognostic risk model based on methionine metabolism-related genes in diffuse large B-cell lymphoma（DLBCL）, investigate its prognostic significance, and clarify the regulatory role of abnormal methionine metabolism in DLBCL progression.

**Methods:**

Clinical prognostic information and methionine metabolism-related gene expression data of patients with DLBCL were obtained from the GEO dataset GSE10846. Lasso regression and Cox regression analyses were performed to screen core prognostic genes and establish a risk model, which was further validated in GSE11318 and GSE181063 datasets. The potential biological functions were analyzed by using Gene Ontology, Kyoto Encyclopedia of Genes and Genomes pathway analysis, and Gene Set Enrichment Analysis. The DLBCL cell lines U2932 and SUDHL-4 were cultured in vitro and divided into the normal culture group（control group）, methionine deprivation group, methionine deprivation + methionine supplementation group（methionine supplementation group）, and methionine deprivation + S-adenosylmethionine（SAM）supplementation group（SAM supplementation group）. Intracellular concentrations of methionine and SAM were detected by liquid chromatography-mass spectrometry. Cell proliferation and apoptosis were measured by using CCK-8 assay and flow cytometry, respectively. A U2932 cell-derived xenograft model was established in NOD-SCID mice, which were assigned to the control diet group（*n*＝5）and methionine-restricted diet group（*n*＝5）, and tumor growth was monitored dynamically.

**Results:**

Eight core genes were screened from 33 methionine metabolism-related genes to construct the prognostic risk model. Kaplan-Meier analysis in the training set revealed that overall survival was significantly shorter in the high-risk group than in the low-risk group（*HR*＝2.50, 95％ *CI*: 1.84–3.39, *P*<0.001）. Stable prognostic performance was confirmed in two validation sets: GSE11318（*HR*＝1.89, 95％ *CI*: 1.30–2.75, *P*<0.001）and GSE181063（*HR*＝1.39, 95％ *CI*: 1.20–1.62, *P*<0.001）. In vitro, compared with the normal culture group, the methionine deprivation group showed significantly decreased intracellular levels of methionine and SAM（both *P*<0.001）, completely inhibited cell proliferation, and increased apoptosis（both *P*<0.001）; compared with the methionine deprivation group, the methionine supplementation group and SAM supplementation group exhibited significantly restored apoptosis（all *P* value<0.001）. In vivo, methionine restriction evidently reduced tumor volume［（438.3±179.1）mm³ *vs*（1 451.0±464.9）mm³, *P*＝0.002］and tumor weight［（0.3±0.1）g *vs*（1.1±0.4）g, *P*＝0.004］compared with the control diet.

**Conclusion:**

The prognostic risk model based on methionine metabolism-related genes shows potential prognostic value for DLBCL. Abnormal methionine metabolism regulates the proliferation and apoptosis of DLBCL cells, providing experimental evidence for the intervention targeting methionine metabolism in DLBCL.

弥漫大B细胞淋巴瘤（DLBCL）是最常见的淋巴瘤，占非霍奇金淋巴瘤的30％～40％，尽管R-CHOP（利妥昔单抗+环磷酰胺+阿霉素+长春新碱+泼尼松）免疫化学治疗方案显著改善DLBCL患者的生存，但仍有近1/3的患者出现疾病复发或原发性耐药[1]–[3]。

蛋氨酸是人体必需的含硫氨基酸，参与蛋白质合成、DNA甲基化以及多胺合成等关键生物学过程，对维持细胞稳态至关重要[4]。研究发现，肿瘤细胞对蛋氨酸的需求显著高于正常细胞[5]。靶向蛋氨酸代谢通路的干预策略，包括蛋氨酸剥夺、代谢酶抑制剂等，在临床前研究中展现出良好的抗肿瘤效应[6]–[8]。然而，蛋氨酸代谢异常在DLBCL进展中的调控作用及其临床预后价值尚未明确。本研究通过构建基于蛋氨酸代谢基因的预后风险模型，同时结合体内外实验，系统探究蛋氨酸依赖性在DLBCL进展中的作用，旨在为DLBCL患者提供新型预后评估工具，并为靶向蛋氨酸代谢的治疗策略提供实验依据与理论支撑。

## 材料与方法

1. 数据来源：从GEO数据库（http://www.ncbi.nlm.nih.gov/geo/）下载GSE10846数据集为训练集，GSE11318和GSE181063为验证集，分别包含412例、200例和1 310例包含生存信息的DLBCL样本。

2. 生物信息学分析：从分子特征数据库（MSigDB）下载蛋氨酸代谢相关基因，包括蛋氨酸和半胱氨酸分解代谢（WP_CYSTEINE_AND_METHIONINE_CATABOLISM）以及蛋氨酸从头和挽救途径（WP_METHIONINE_DE_NOVO_AND_SALVAGE_PATHWAY）基因集。去除重叠基因后获得33个蛋氨酸代谢相关基因。使用R语言“survival”包进行单因素Cox回归分析及PH检验，通过“glmnet”包对交集基因进行Lasso回归分析，筛选预后基因。

根据所筛选的基因和系数构建预后风险模型：风险评分＝$\overset{n}{\underset{i=0}{\sum }}\left(\beta i*\chi i\right)$（*βi*为各基因的权重系数，*χi*为各基因的表达量）。将风险评分从低到高进行排序，以中位数为界将患者分为高风险组和低风险组。使用R语言“survival”和“survminer”包对总生存（OS）期进行分析，并使用“timeROC”包绘制1、3、5年生存受试者工作特征（ROC）曲线及计算曲线下面积（AUC）值。使用“limma”包进行差异表达基因分析，“clusterProfiler”包进行基因本体（GO）、京都基因与基因组百科全书（KEGG）通路分析和基因集富集分析（GSEA）。

3. 细胞系和培养条件：DLBCL细胞株U2932和SUDHL-4购自中乔新舟生物科技有限公司（中国上海），并通过短串联重复序列（STR）鉴定。细胞用含有10％胎牛血清（南京生航生物技术有限公司）和1％青霉素-链霉素的RPMI1640培养基于37 °C、5％ CO_2_条件下培养。蛋氨酸剥夺处理指细胞使用不含蛋氨酸的RPMI1640培养基（美国Gibco公司，货号：A1451701）进行培养，其他成分均一致。

4. CCK-8检测细胞活力：取对数生长期DLBCL细胞，分为两组，分别使用正常对照培养基和蛋氨酸剥夺培养基进行培养，即正常对照组和蛋氨酸剥夺组。将细胞以1×10^5^/ml密度接种于96孔板，每组3个复孔，每孔加入10 µl CCK-8试剂，避光孵育3 h，酶标仪读取450 nm处各孔吸光度（*A*）值。连续检测第0～3天*A*值，计算细胞增殖活力：（*A*_实验组_−*A*_空白组_）/（*A*_对照组_−*A*_空白组_）×100％。

5. 流式细胞术检测细胞凋亡：对细胞进行蛋氨酸剥夺处理，或同时补充100 µmol/L蛋氨酸（美国Sigma公司，货号：M5308）或100 µmol/L S-腺苷蛋氨酸（SAM）（美国Sigma公司，货号：A4377），分为4组：正常培养组（对照组）、蛋氨酸剥夺组（Met剥夺组）、蛋氨酸剥夺+蛋氨酸补充组（Met补充组）、蛋氨酸剥夺+SAM补充组（SAM补充组），培养48 h后收集细胞。根据细胞凋亡检测试剂盒（杭州联科生物技术有限公司，货号：AP105）说明书对细胞进行凋亡检测，每组设置3个生物学重复。其中蛋氨酸和SAM均用无酶H_2_O进行溶解。

6. 实时聚合酶链反应（RT-PCR）：收集对照组和Met剥夺组细胞，使用Trizol（美国Invitrogen公司）法提取RNA。按照说明书体系逆转录合成cDNA（南京诺唯赞生物科技股份有限公司，货号：R323-01），RT-PCR检测按照Taq Pro Universal SYBR qPCR Master Mix说明书进行操作（南京诺唯赞生物科技股份有限公司，货号：Q712-02）。采用2^−ΔΔCt^法计算目标基因的相对表达量，ΔCt＝目标基因Ct值−内参基因Ct值，ΔΔCt＝处理组ΔCt值−对照组ΔCt值。目标基因及内参基因引物由OriGene网站（https://www.origene.com.cn/）获得并经Primer-BLAST验证，由南京擎科生物科技有限公司合成。具体序列见附表1（**扫描本文首页二维码查阅**）。

7. 液相色谱-串联质谱（LC-MS/MS）检测分析胞内蛋氨酸、SAM浓度：收集对照组、Met剥夺组、Met补充组、SAM补充组细胞，细胞破碎后，向细胞沉淀中加入300 µl甲醇进行稀释，超声处理10 min，然后涡旋混匀10 min。4 °C，19 000 ×*g*离心10 min，取上清进行LC-MS/MS分析。每组设置3个生物学重复。该分析由苏州沪云新药研发股份有限公司协助。

8. 动物实验：将PBS重悬的DLBCL细胞系U2932细胞与基质胶以1∶1的体积比混合后将其皮下注射于6～8周龄的NOD-SCID小鼠前肢腋窝（1×10^7^个细胞/只）。小鼠由江苏集萃药康生物科技股份有限公司提供，并饲养于无特殊病原体（SPF）级实验动物中心。移植后7 d左右小鼠成瘤。将荷瘤小鼠随机分为2组，每组5只。分别给予热量相同的正常饲料和蛋氨酸缺乏饲料（江苏省协同医药生物工程有限责任公司），当天定义为第0天。连续喂养并监测1个月，每隔3 d测量并计算小鼠的肿瘤体积。小鼠肿瘤体积若达到伦理限制，将提前对其进行安乐死。本研究经南京医科大学实验动物伦理委员会批准（批件号：IACUC512098）。

9. 统计学处理：使用Graphpad prism 9.5及R 4.5.0软件进行数据处理和分析。数据以*x*±*s*表示，两组间比较采用Student's *t*检验，多组间比较采用单因素方差分析，每个实验至少重复3次，以*P*<0.05为差异有统计学意义。

## 结果

1. 蛋氨酸代谢风险评分预后模型构建：从分子特征数据库（MSigDB）中获取33个与蛋氨酸代谢相关的基因（**扫描本文首页二维码查阅附图1A**），基于这些基因对GSE10846数据集中的DLBCL样本进行单因素Cox回归分析，筛选出17个与生存显著相关的基因（*P*值均<0.05，**扫描本文首页二维码查阅附图1B**）。将上述基因纳入Lasso回归模型分析，通过确定最佳回归系数及交叉验证，最终筛选8个基因作为预后模型核心基因（**扫描本文首页二维码查阅附图1C、D**）。根据各基因表达量与风险系数为DLBCL患者计算风险评分，公式为：风险评分＝0.254×SRM基因表达量+0.178×MAT2A基因表达量+0.224×MPST基因表达量+（−0.027）×MSRB2基因表达量+0.216×GCLC基因表达量+0.289×GSS基因表达量+0.218×AMD1基因表达量+0.154×TXN基因表达量。以风险评分中位数为界将患者分为高、低风险组，其中MSRB2为总生存（OS）的保护因素，SRM、MAT2A、MPST、GCLC、GSS、AMD1及TXN 7个基因为危险因素（**扫描本文首页二维码查阅附图1E、F**）。采用时间依赖性ROC曲线分析模型预后预测的敏感性与特异性，结果显示其预测1年、3年、5年生存率的AUC值分别为0.69、0.69、0.68（**扫描本文首页二维码查阅附图1G**）。Kaplan-Meier曲线显示，高风险组患者生存情况较低风险组差（*HR*＝2.50，95％ *CI*：1.84～3.39，*P*<0.001，**扫描本文首页二维码查阅附图1H**）。验证集（GSE11318、GSE181063）结果进一步证实，患者蛋氨酸代谢预后风险评分越高，预后越差（GSE11318：*HR*＝1.89，95％ *CI*：1.30～2.75，*P*<0.001；GSE181063：*HR*＝1.39，95％ *CI*：1.20～1.62，*P*<0.001，**扫描本文首页二维码查阅附图1I、J**）。

2. 蛋氨酸代谢预后模型通路富集分析：以|log2表达倍数|>0.5且*P*值<0.05为筛选条件，对高、低风险组间的差异表达基因进行分析，共鉴定出4 041个差异表达基因。其中上调表达基因2 705个，下调表达基因1 336个。为探讨差异基因的分子功能及作用机制，分别进行了GO、KEGG和GSEA分析。GO结果显示，高风险组差异基因在DNA复制、细胞生长等通路上明显富集（图1A），KEGG分析主要富集在PI3K-AKT等致癌信号通路（图1B）。GSEA结果同样表明，两组在MYC靶点通路等多个调控细胞增殖的关键生物学功能及信号通路中存在显著差异（图1C）。

**图1:**
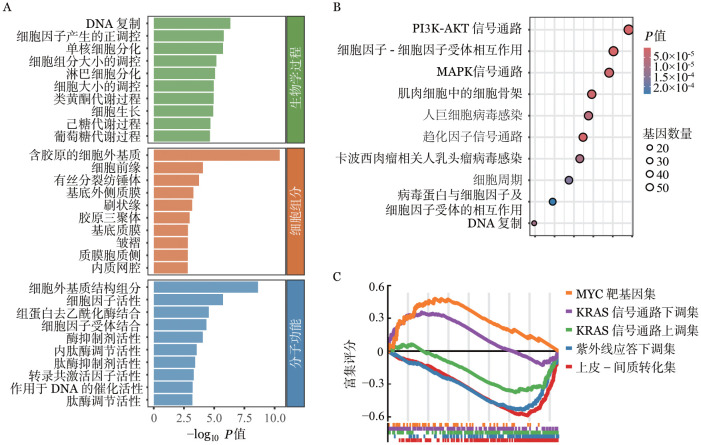
蛋氨酸代谢预后模型高、低风险组差异基因通路富集分析 **A** 高、低风险组之间差异基因的基因本体（GO）分析；**B** 高、低风险组之间差异基因的京都基因与基因组百科全书（KEGG）通路分析；**C** 高、低风险组之间差异基因的基因集富集分析（GSEA）

3. DLBCL细胞存活依赖蛋氨酸：为明确DLBCL细胞对蛋氨酸的依赖性，对U2932和SUDHL-4细胞进行蛋氨酸剥夺处理，结果显示蛋氨酸剥夺可完全抑制细胞增殖（U2932细胞相对活力：第0天：1.00±0.04对1.00±0.02，*P*>0.05；第1天：1.75±0.11对2.38±0.20，*P*<0.001；第2天：1.89±0.09对7.88±0.41，*P*<0.001；第3天：1.89±0.30对8.70±0.55，*P*<0.001；SUDHL-4细胞相对活力：第0天：1.00±0.02对1.00±0.095，*P*>0.05；第1天：1.82±0.04对2.33±0.22，*P*＝0.016；第2天：1.89±0.07对4.67±0.14，*P*<0.001；第3天：1.87±0.12对7.68±0.08，*P*<0.001）。代谢物检测及凋亡检测结果显示（表1）：与正常培养组（对照组）相比，蛋氨酸剥夺处理后（Met剥夺组），两种DLBCL细胞系内蛋氨酸浓度均降低，下游代谢产物SAM水平亦下降，同时细胞凋亡率升高；而在蛋氨酸剥夺的同时外源性补充蛋氨酸（Met补充组），细胞内蛋氨酸及SAM浓度均明显回升，凋亡水平降低（*P*值均<0.001）。此外，外源性补充SAM（SAM补充组）也可部分逆转蛋氨酸缺乏引起的细胞凋亡（表1）。通过RT-qPCR检测蛋氨酸代谢通路8个核心基因的mRNA表达水平发现，蛋氨酸剥夺后，U2932和SUDHL-4细胞中MAT2A的mRNA相对表达量均呈明显上调趋势（U2932：4.84±0.07对1.00±0.02，*P*<0.001；SUDHL-4：5.44±0.46对1.00±0.04，*P*<0.001）。

**表1:** 不同干预条件下弥漫大B细胞淋巴瘤细胞系内蛋氨酸、SAM含量及细胞凋亡率变化（*x*±*s*）

组别	U2932			SUDHL-4		
	细胞内蛋氨酸含量（nmol/10^6^个细胞）	细胞内SAM含量（nmol/10^6^个细胞）	细胞凋亡率（%）	细胞内蛋氨酸含量（nmol/10^6^个细胞）	细胞内SAM含量（nmol/10^6^个细胞）	细胞凋亡率（%）
对照组	498.39±5.63	78.99±0.89	9.98±0.53	285.01±4.54	63.02±1.00	11.95±2.61
Met剥夺组	203.87±5.42	8.26±0.22	89.96±0.19	97.49±1.77	8.04±0.07	81.33±1.39
Met补充组	608.96±7.76	54.67±0.70	45.37±0.98	250.90±2.48	47.56±0.45	17.79±0.25
SAM补充组	190.88±3.80	46.71±0.13	72.79±1.29	74.23±1.45	29.81±0.29	62.48±2.78

*P*1值	<0.001	<0.001	<0.001	<0.001	<0.001	<0.001
*P*2值	<0.001	<0.001	<0.001	<0.001	<0.001	<0.001
*P*3值	/	<0.001	<0.001	/	<0.001	<0.001

**注** SAM：S-腺苷蛋氨酸；对照组：正常培养组；Met剥夺组：蛋氨酸剥夺组；Met补充组：蛋氨酸剥夺+蛋氨酸补充组；SAM补充组：蛋氨酸剥夺+SAM补充组；*P*1：对照组与Met剥夺组比较；*P*2：Met剥夺组与Met补充组比较；*P*3：Met剥夺组与SAM补充组比较；/：未进行统计学比较

4. 蛋氨酸限制抑制DLBCL肿瘤进展：为评估蛋氨酸在体内对DLBCL肿瘤进展的影响，将U2932细胞移植至NOD-SCID小鼠体内构建DLBCL荷瘤模型，随后分别给予常规饮食（对照组）和蛋氨酸限制性饮食（Met剥夺组）干预。结果显示，Met剥夺组小鼠的肿瘤生长受到明显抑制（图2A）；终点检测显示，与对照组相比，Met剥夺组小鼠的肿瘤体积［（438.3±179.1）mm^3^对（1 451.0±464.9）mm^3^，*P*＝0.002，图2B］和肿瘤重量［（0.3±0.1）g对（1.1±0.4）g，*P*＝0.004，图2C］均降低，证实蛋氨酸剥夺具有体内抗DLBCL作用。值得注意的是，整个实验期间，两组小鼠均能耐受相应饮食，Met剥夺组小鼠未出现明显精神状态异常，但体重显著低于对照组（*P*＝0.002，图2D）。

**图2:**
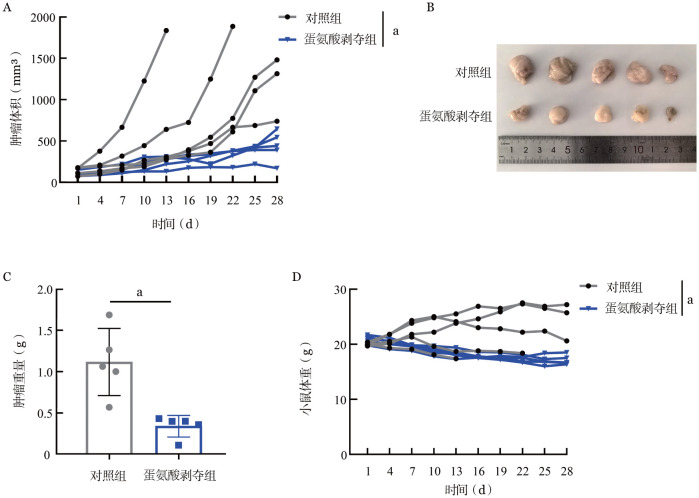
蛋氨酸限制抑制弥漫大B细胞淋巴瘤荷瘤小鼠肿瘤进展 **A** 蛋氨酸限制性饮食（蛋氨酸剥夺组，5只）与常规饮食（对照组，5只）小鼠肿瘤生长体积变化；**B** 两组小鼠肿瘤大小（肿瘤于处死小鼠后分离并拍照）；**C** 两组小鼠肿瘤重量比较；**D** 两组小鼠体重变化 **注** ^a^*P*<0.01

## 讨论

蛋氨酸作为人体必需的含硫氨基酸，在肿瘤发生发展中具有重要意义。与正常细胞相比，肿瘤细胞对蛋氨酸表现出显著的代谢依赖性，这一特性与其独特的代谢重编程特征相关。限制膳食中蛋氨酸已成为一种极具潜力的癌症治疗策略[9]。在急性髓系白血病（AML）中，蛋氨酸缺乏可有效抑制疾病进展[6]。在EB病毒（EBV）相关伯基特淋巴瘤中，干扰B细胞对蛋氨酸的摄取或代谢，不仅可改变肿瘤细胞内EBV基因组程序，还能抑制EBV介导的原发性B细胞生长转化[10]。然而，蛋氨酸在DLBCL中的作用仍不明确。阐明DLBCL细胞对蛋氨酸的依赖性，有助于更深入地理解疾病进展，并为挖掘潜在的可靶向代谢靶点、开发新型治疗干预策略提供重要依据。

本研究从蛋氨酸代谢相关基因中筛选出8个核心预后基因，以此构建DLBCL预后风险模型。该模型在训练集及2个验证集中均表现出稳定的预后预测能力，高风险组患者的OS期显著短于低风险组，提示其可作为临床新型预后评估工具。进一步的功能富集分析显示，高风险组差异基因主要富集于DNA复制、细胞生长等生物学过程，以及PI3K-AKT信号通路、MYC靶点通路等致癌通路。PI3K-AKT通路作为DLBCL中高频激活的信号通路，通过调控糖代谢、脂质代谢及细胞周期进程促进肿瘤进展[11]–[12]；MYC基因扩增或过表达是DLBCL的重要分子特征，其靶基因参与细胞增殖、代谢重编程等关键过程[13]–[15]。这一结果提示，蛋氨酸代谢异常可能与PI3K-AKT、MYC等致癌信号通路相互作用，共同驱动DLBCL的恶性进展，为阐明高风险组患者预后不良的分子机制提供了重要线索。

正常细胞能够通过自身的代谢调节适应蛋氨酸缺乏，而肿瘤细胞因代谢重编程，过度依赖外源性蛋氨酸以满足快速增殖和恶性存活的需求。多项研究表明，蛋氨酸对维持肿瘤生长至关重要。限制饮食中的蛋氨酸可以抑制食管鳞癌、乳腺癌、胶质瘤等肿瘤进展[16]–[18]。本研究发现，DLBCL细胞的存活显著依赖于蛋氨酸。蛋氨酸剥夺可导致细胞内蛋氨酸及下游代谢产物SAM浓度显著下降，进而抑制细胞增殖并促进凋亡；而外源性补充蛋氨酸或SAM则能够逆转上述效应。值得注意的是，蛋氨酸剥夺后，DLBCL细胞中蛋氨酸代谢通路关键酶MAT2A mRNA水平明显上调。这一适应性表达变化提示DLBCL细胞对蛋氨酸代谢的高度依赖，即细胞试图通过增强核心代谢酶的表达，维持蛋氨酸代谢通路功能及SAM生成，以缓解甲基供体不足带来的代谢压力。以上结果为开发靶向蛋氨酸代谢的治疗策略提供直接实验依据。然而，蛋氨酸或SAM补充虽能部分逆转蛋氨酸剥夺诱导的DLBCL细胞凋亡，但未完全恢复至对照组水平，且不同亚型DLBCL细胞对SAM补充的应答存在明显差异。这一现象可能与DLBCL细胞的亚型特异性代谢特征、固有凋亡通路差异密切相关[19]。此外，体内实验进一步证明，蛋氨酸缺乏饮食可显著抑制DLBCL荷瘤小鼠的肿瘤生长，但同时导致了小鼠体重下降。这一结果提示，尽管蛋氨酸剥夺具有明确的抗肿瘤作用，但蛋氨酸作为正常细胞必需的营养物质，非特异性蛋氨酸限制可能对正常组织造成损伤，影响治疗耐受性。因此，未来需要探索更具特异性的干预策略，例如，靶向蛋氨酸代谢通路关键酶的小分子抑制剂，在精准抑制肿瘤细胞蛋氨酸代谢的同时，避免影响正常细胞的营养供应；或将蛋氨酸限制与化疗、免疫治疗等传统治疗手段联合应用，减少治疗剂量相关不良反应，从而进一步提升DLBCL患者的治疗获益。

尽管本研究为DLBCL的预后评估与代谢靶向治疗提供了线索，但仍存在一定的局限性：预后模型的构建与验证均基于公共数据集，缺乏前瞻性临床队列的验证，其在不同临床场景及治疗背景下的适用性仍需进一步探索；体内实验采用的是免疫缺陷小鼠荷瘤模型，未能模拟人体复杂的肿瘤微环境及免疫系统作用，可能在一定程度上影响结果的临床转化价值；本研究仅初步证实蛋氨酸代谢异常与DLBCL进展相关，但对蛋氨酸代谢与PI3K-AKT、MYC等致癌信号通路之间的具体相互作用机制尚未完全阐明。

综上所述，本研究构建的蛋氨酸代谢预后风险模型为DLBCL患者提供了一种新型、可靠的预后评估工具，具有辅助临床预后分层与治疗决策的潜力。同时，研究证实蛋氨酸依赖性是DLBCL进展的关键代谢特征，靶向蛋氨酸代谢通路可能成为DLBCL患者的潜在治疗策略。未来需通过前瞻性临床研究验证模型的可靠性，扩大细胞株及动物模型的覆盖范围，并深入解析蛋氨酸代谢异常的分子机制，有望优化预后模型并开发特异性干预药物，从而进一步提高DLBCL的治疗效果，改善患者预后。

## Supplementary Material


