# Supplementary figures and images for: Construction of a methionine metabolism-related prognostic signature and the effect of targeting methionine metabolism on diffuse large B-cell lymphoma
Source: Zhonghua Xue Ye Xue Za Zhi. 2026 May;47(5):473–9. [Article in Chinese] doi: 10.3760/cma.j.cn121090-20251219-00603 (PMC13416638; doi:10.3760/cma.j.cn121090-20251219-00603)

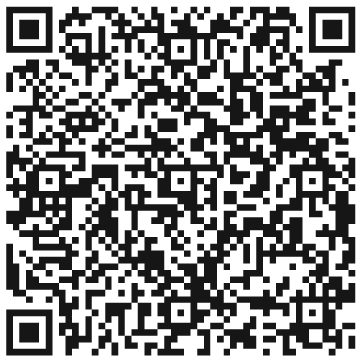

Supplement: Supplementary file 1 [file cjh-47-05-473-g003.tif]
